# Human renal response to furosemide: Simultaneous oxygenation and perfusion measurements in cortex and medulla

**DOI:** 10.1111/apha.13292

**Published:** 2019-05-21

**Authors:** Bryan Haddock, Henrik B. W. Larsson, Susan Francis, Ulrik B. Andersen

**Affiliations:** ^1^ Department of Clinical Physiology, Nuclear Medicine & PET Rigshospitalet, Copenhagen University Hospital Glostrup Denmark; ^2^ Sir Peter Mansfield Magnetic Resonance Centre School of Physics and Astronomy University of Nottingham Nottingham UK

**Keywords:** ASL, BOLD, hypertension, kidney, MRI, RBF, renal

## Abstract

**Aim:**

Disturbances of renal medullary perfusion and metabolism have been implicated in the pathogenesis of kidney disease and hypertension. Furosemide, a loop diuretic, is widely used to prevent renal medullary hypoxia in acute kidney disease by uncoupling sodium metabolism, but its effects on medullary perfusion in humans are unknown. We performed quantitative imaging of both renal perfusion and oxygenation using Magnetic Resonance Imaging (MRI) before and during furosemide. Based on the literature, we hypothesized that furosemide would increase medullary oxygenation, decrease medullary perfusion, but cause minor changes (<10%) in renal artery flow (RAF).

**Methods:**

Interleaved measurements of RAF, oxygenation (*T*_2_*) and perfusion by arterial spin labelling in the renal cortex and medulla of 9 healthy subjects were acquired before and after an injection of 20 mg furosemide. They were preceded by measurements made during isometric exercise (5 minutes handgrip bouts), which are known to induce changes in renal hemodynamics, that served as a control for the sensitivity of the hemodynamic MRI measurements. Experiments were repeated on a second day to establish that the measurements and the induced changes were reproducible.

**Results:**

After furosemide, *T*_2_* values in the medulla increased by 53% (*P* < 0.01) while RAF and perfusion remained constant. After hand‐grip exercise, *T*_2_* values in renal medulla increased by 22% ± 9% despite a drop in medullary perfusion of 7.2% ± 4.7% and a decrease in renal arterial flow of 17.5% ± 1.7% (*P* < 0.05). Mean coefficients of variation between repeated measurements for all parameters were 7%.

**Conclusion:**

Furosemide induced the anticipated increase in renal medullary oxygenation, attributable exclusively to a decrease in renal oxygen consumption, since no change of RAF, cortical or medullary perfusion could be demonstrated. All measures and the induced changes were reproducible.

## 1 INTRODUCTION

The renal medulla has a complex structure of parallel tubules and vessels running from the cortex to the papillae and back, enabling the concentration of urine. The combination of low flow and high metabolism in this system creates a very low oxygen tension, on the edge of hypoxia. Alterations in perfusion or metabolic rate which further reduce medullary oxygenation has been implicated in the pathogenesis of acute kidney disease (acute tubulointerstitial nephropathy),^1^ but also some forms of chronic kidney disease,^2^ essential hypertension^3^ and hypertension secondary to other causes, such as renal artery stenosis.^4^

Reliable and non‐invasive methods to study these mechanisms in the human kidney are therefore mandatory, but have not been accessible until recently.^5^, ^6^, ^7^, ^8^, ^9^ Renal blood oxygenation can be monitored with Magnetic Resonance Imaging (MRI) using the paramagnetic property of deoxyhemoglobin as a surrogate measure using the blood oxygenation level‐dependent contrast (BOLD). The BOLD imaging signal is expressed by the transverse relaxation time (*T*_2_*) or rate (*R*_2_* = 1/*T*_2_*). Renal tissue perfusion can be measured noninvasively with MRI using arterial spin labelling (ASL), whilst phase contrast (PC) MRI can be used to measure renal artery flow (RAF). Changes in the human renal cortex and medulla oxygenation have been reported by several authors during various manipulations, including furosemide injection.^10^, ^11^ However, tissue oxygenation depends on the net balance between oxygen delivery and oxygen consumption.^9^ *T*_2_* measurements do not differentiate between these factors and, for this reason, it has been suggested that the measurement of renal blood flow is mandatory to interpret renal *T*_2_* values.^12^, ^13^ Recently, hand grip exercise has been demonstrated to induce a similar increase in medullary *T*_2_* values indicating increased blood oxygenation^6^ although renal blood flow and medullary perfusion contradicting common assumptions regarding perfusion and blood oxygenation. Hand grip exercise reduces RAF via a powerful stimulating effect on the renal sympathetic nerve activity.^14^

Furosemide, a loop diuretic, is among the most widely used drugs, being a cornerstone in the treatment of fluid overload in diseases such as heart failure and CKD and previously used to prevent acute tubulointerstitial nephropathy.^15^ Furosemide has its main action as an inhibitor of the sodium‐potassium‐chloride cotransporter (NKCC2) in the tubular epithelium in the thick ascending limb of Henles loop, thus uncoupling the sodium reabsorption in the renal medulla. Inhibition of NKCC2 in the macula densa blunts the expected tubuloglomerular feedback response to the increased distal sodium delivery.^16^, ^17^ It has become clear though, that furosemide has complex systemic and renal effects that may affect renal perfusion as well as metabolism.^18^

The purpose of this study is to determine whether or not the previously reported furosemide induced changes in renal blood oxygenation result from a haemodynamic response. We hypothesise that *T*_2_*, renal perfusion and renal artery blood flow measures will remain constant based on our belief that these changes in blood oxygenation are driven by reductions in metabolism.

## 2 RESULTS

All nine subjects completed the scanning protocol. Physiological monitoring (BP, SaO_2_ and HR) was completed in all but three scan sessions due to technical reasons.

**Table 1:** Measured values and coefficient of variation

	Resting		After furosemide	
	Mean value (all subjects)	CV Day1 to Day 2 (all subjects)	Mean value (all subjects)	CV Day 1 to Day 2 (3 subjects)
*T*_2_* Cort	50 ± 2 ms	4.2%	51 ± 3 ms	4.7%
*T*_2_* Med	23 ± 4 ms	6.4%	35 ± 7 ms*	8.6%
Perf Cort	273 ± 38 mL 100 g^−1^ min^−1^	5.1%	280 ± 41 mL 100 g^−1^ min^−1^	8.4%
Perf Med	38 ± 5 mL 100 g^−1^ min^−1^	11.2%	39 ± 7 mL 100 g^−1^ min^−1^	5.6%
Renal Flow	709 ± 176 mL min^−1^	6.6%	710 ± 155 mL min^−1^	5.8%

**Figure 1:**
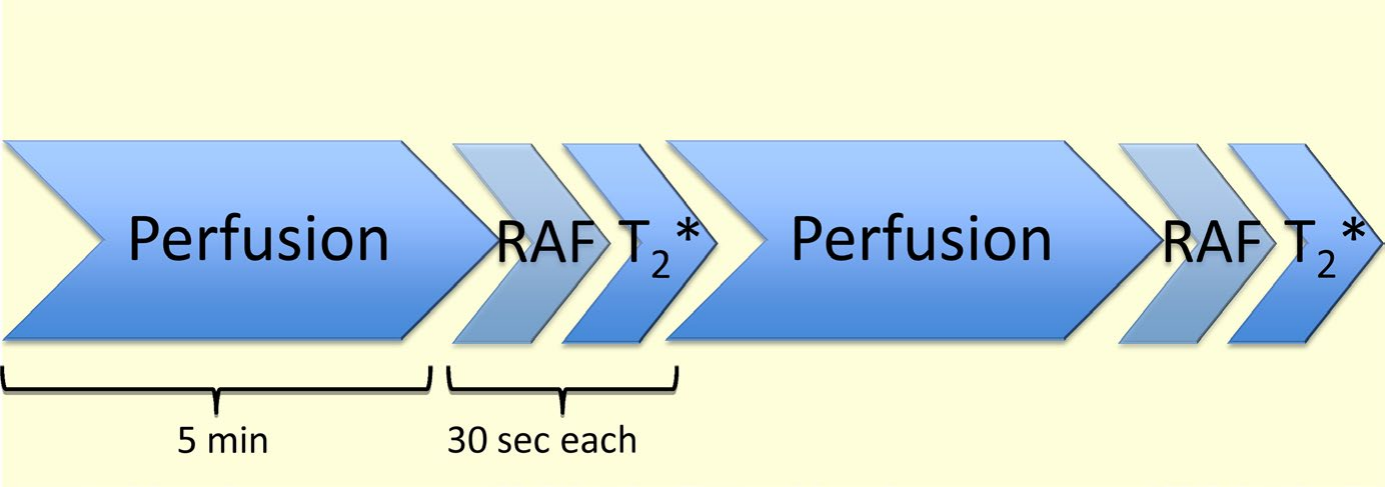
Flowchart of MRI data acquisition. In the MRI session, two data sets were collected for each MRI parameter (perfusion, RAF and *T*_2_*) prior to the injection of furosemide and then again afterwards. Measurements were collected in the order shown in the figure. Total scan time was approximately 45 min

**Figure 2:**
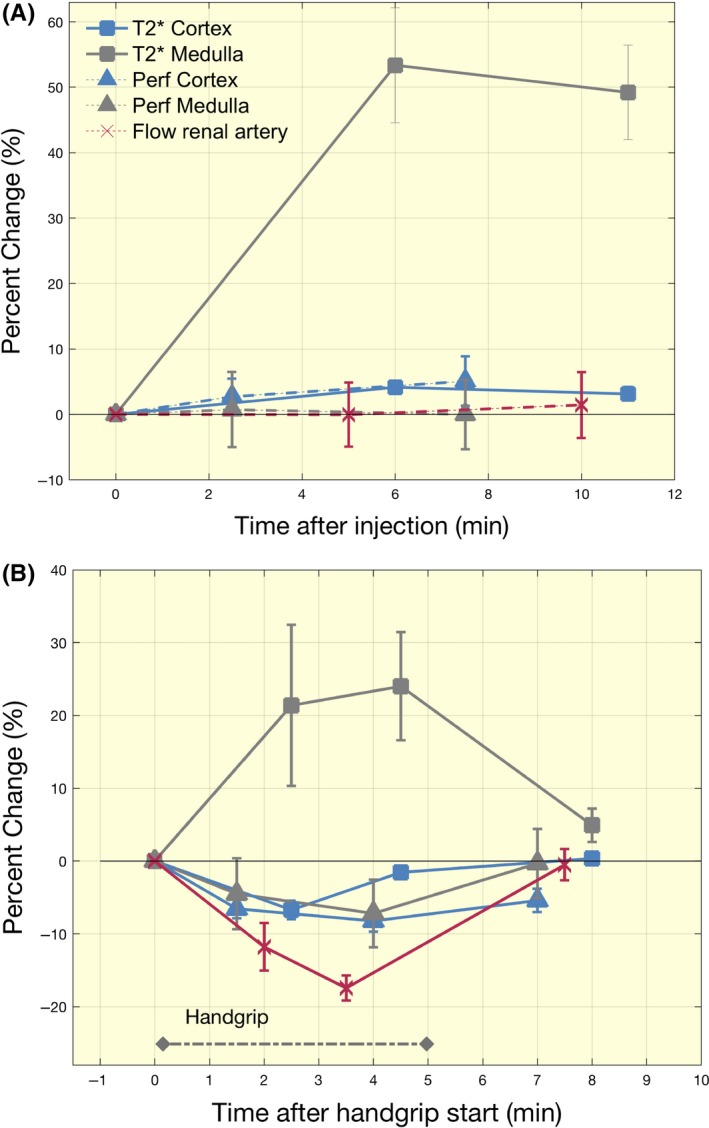
Response to Furosemide. Percent change in MRI parameters of *T*_2_* and perfusion in renal cortex and medulla, and renal artery flow, measured before and after the furosemide injection (A). Only *T*_2_* in the medulla increased significantly (*P* < 0.01) and there were no significant changes between the two post injection time points. Both flow and cortical flow remained constant with changes averaging less than 5%. (B): For comparison, we show changes during the subjects handgrip exercise for the same trials (this handgrip data is included in a larger dataset published in Haddock et al^6^) with renal artery blood flow and perfusion decreasing significantly during the intervention (*P* < 0.05). Error bars are ± SEM. Measures of *T*_2_* and renal artery flow are collected over ~30 s and for perfusion over ~3 min. Time points are centred to the middle of the measurement period

Mean and standard deviation together with coefficient of variation (CV) for BOLD *T*_2_* values of oxygenation, perfusion measured using arterial spin labelling (ASL), and renal artery blood flow (right renal artery) calculated using phase contrast MRI. Subjects were scanned with these MRI techniques on two separate days though only 3 subjects received a second injection of furosemide. Mean values after furosemide include both measures for each parameter between 3 and 12 min post–injection for all subjects. For measurement time points see Figures 1 and 2 (*P < 0.01).

### 2.1 RAF, *T*_2_* and perfusion

**Figure 3:**
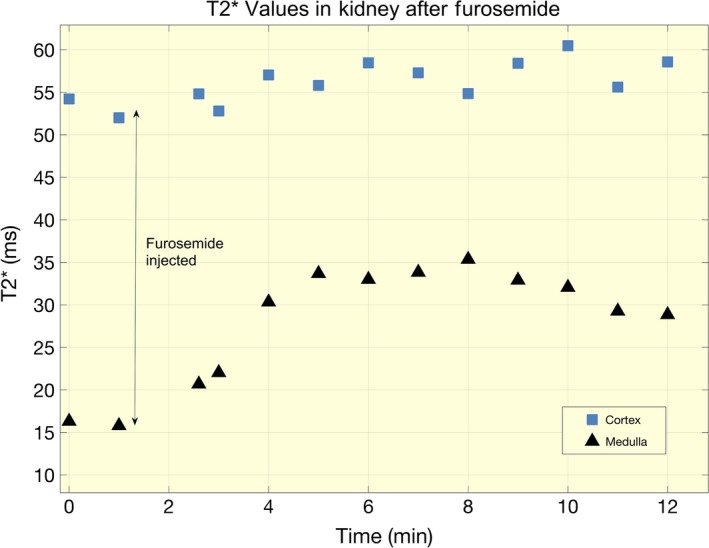
Continuous measurements of *T*_2_*. Absolute *T*_2_* measures in a single subject before and after injection of furosemide

Following the injection of furosemide, a significant increase in *T*_2_* (*P* < 0.001) was observed in the medulla (group average resting *T*_2_* value of 22.3 ± 2.6 ms increasing to 35 ± 2.6 ms on furosemide). In the renal cortex, mean *T*_2_* values increased slightly from 49.5 ± 2.0 ms to 51.3 ± 3.0 ms No significant changes in perfusion in the medulla or cortex were noted, nor were there any significant changes in RAF. Relative changes in all parameters are shown in Figure 2, and mean results from all MRI measurements with CVs between Day 1 and Day 2 are presented in Table 1. Data from an extra trial from one subject showing changes in renal *T*_2_* each minute after the furosemide injection is also presented in Figure 3. After four minutes of handgrip exercise, perfusion decreased (*P* < 0.05) by −8.2% ± 1.% and −7.2% ± 4.7% in the cortex and medulla, respectively, whereas RAF decreased by −17.5% ± 1.7%. *T*_2_* increased in the medulla by 24.0 7.4% (*P* < 0.01) as opposed to no significant change in the cortex (Figure 2).

### 2.2 Reproducibility

**Figure 4:**
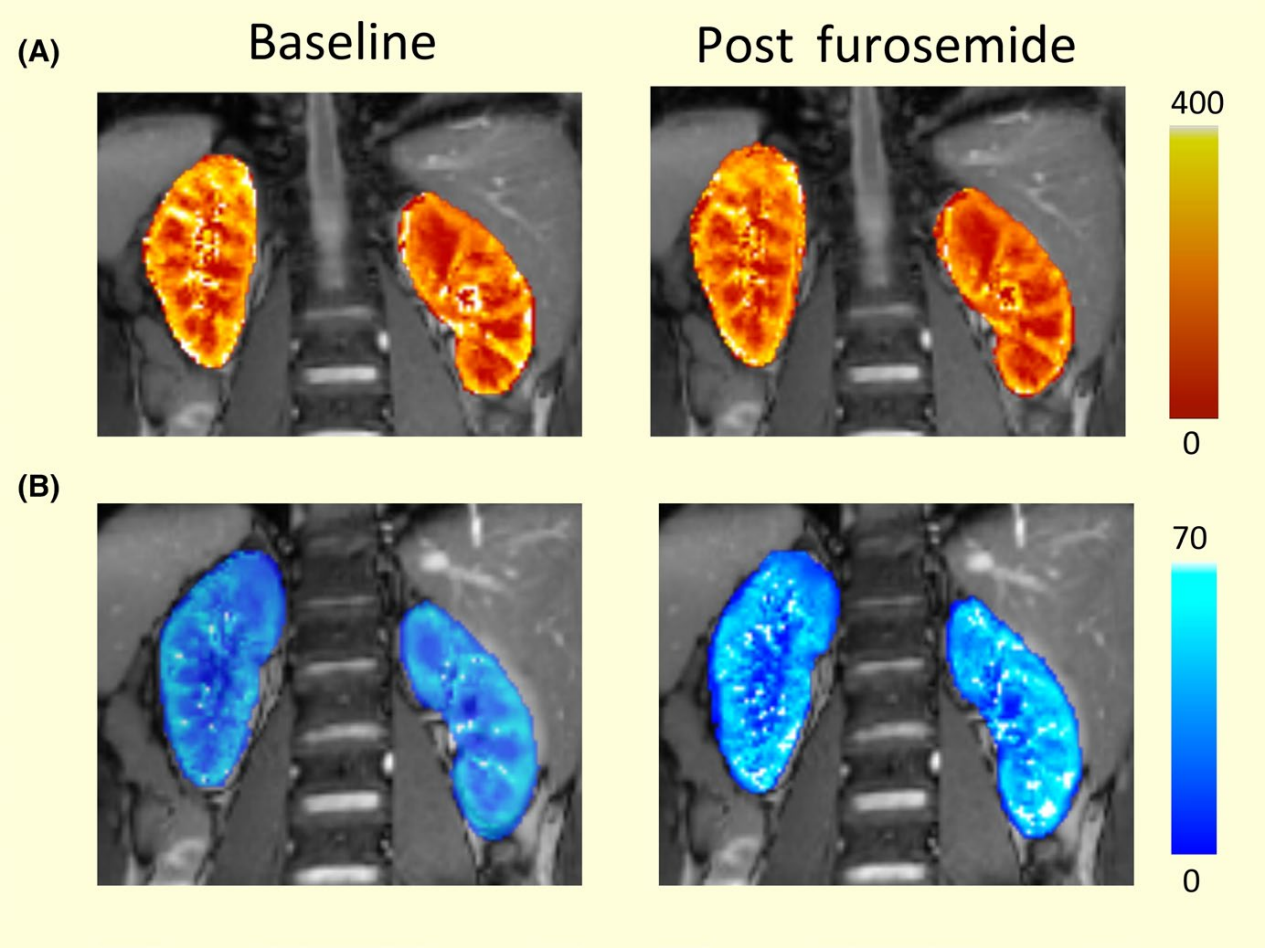
* T*_2_* and perfusion maps from one subject. Perfusion maps A) in mL 100 g^−1^ min^−1^ obtained from one subject during a single trial at baseline and after furosemide injection and B) *T*_2_* maps in ms for the same subject. There were no significant changes in medullary or cortical perfusion, whilst there was a significant increase in medullary *T*_2_*, reducing the corticomedullary contrasts

Resting measurements of *T*_2_* and renal artery blood flow were consistent between Day 1 and Day 2 with CV values of less than 6.5%. Perfusion measurements ranged from 230 to 343 mL 100 ×g^−1^ min^−1^ in the cortex, 34‐50 mL 100 ×g^−1^ min^−1^ in the medulla with CV values of 5% and 11%, respectively, between Day 1 and Day 2. An example perfusion map from one subject's Day 1 scan is shown in Figure 4. Renal artery blood flow ranged between 3.6 and 7.3 mL s^−1^ with a reproducibility CV of 6.6%. Lastly, *T*_2_* values ranged from 45 to 53 ms in the cortex and 18 to 26 ms in the medulla, with CV values of 4.2% and 6.4%, respectively.

### 2.3 Hemodynamics

Blood pressure, oxygen saturation and pulse remained constant after injection of furosemide (Table 2). Group average blood pressure and oxygen saturation remained within 1% of preinjection values.

**Table 2:** Physiological parameters

	2 min prior to injection	0‐5 min	6‐10 min
Blood pressure (mmHg)	131/73 ± 9/8	131/72 ± 8/8	132/73 ± 10/8
Heart rate (bpm)	56 ± 6	55 ± 6	56 ± 7
SaO2 (%)	97.8 ± 0.9	97.7 ± 0.9	97.7 ± 0.6

Mean values of blood pressure, heart rate and blood saturation (SaO_2_) in all subjects prior to and at intervals after injection of furosemide. There were no significant changes in any of the three parameters after furosemide injection.

## 3 DISCUSSION

In this study, we demonstrate the applicability and reproducibility of a combined multiparametric MRI protocol to concurrently measure variations in renal artery blood flow, and oxygenation (*T*_2_*) and perfusion (ASL) in the renal cortex and medulla during an intervention. The combination of these interleaved measurements enables an improved interpretation regarding oxygen consumption in the kidney. After the injection of furosemide, a significant increase in medullary *T*_2_* was observed in the renal medulla, while renal artery blood flow and perfusion were unchanged. This suggests that changes in *T*_2_* are not the result of increased perfusion, but likely due to a decrease in metabolism caused by the uncoupling of sodium transport in the loop of Henle. In contrast, in our previously published study with renal sympathetic activation elicited by handgrip exercise,^6^ *T*_2_* increased in the medulla despite a decrease in medullary perfusion. We interpret this increase as being due to reduced metabolism caused by reduced renal artery blood flow and hence reduced distal sodium delivery.

Values obtained for baseline parameters are in good agreement with values reported in earlier studies. Cortical perfusion has previously been measured to be 321,^19^ 278 mL 100 ×g^−1^ min^−1^,^20^ and 254 mL 100 ×g^−1^ min^−1^ using Gd contrast^21^ which compares well with our group mean of 273 mL 100g^−1^ min^−1^. Other MR‐based approaches have also reported similar results, with values of 339 mL 100g^−1^ min^−1^ using iron oxide nanoparticles.^22^ ASL was chosen for this study due to its ability to provide repeated measurements, not possible using methods requiring contrast or invasive procedures. We find a difference between cortical and medullary *T*_2_* with a mean ratio of 2.4, in agreement with two other 3T studies in healthy subjects^23^, ^24^ which have identical water abstinence periods before the study, while subjects in the study by Pruijm et al^25^ were hydrated. After furosemide, *T*_2_* was unchanged in the cortex but increased in the medulla, reaching a maximum value after approximately 5 minutes, which is comparable to the findings of Tumkur et al^23^ In this study, the mean cortical/medullary *T*_2_* ratio fell to 1.5 after furosemide injection. Repeated examinations on separate days showed good reproducibility as expressed by low CV values for all parameters. This includes the response to furosemide in subjects having received an injection on both days. Two recent studies have found similar reproducibility when measuring renal artery blood flow with phase contrast MRI (CV of 8%‐9%),^26^, ^27^ though earlier studies have experienced larger variation.^28^ Likewise, cortical perfusion and *T*_2_* measurements also had reproducibility values similar to or lower than CV values reported in the recent literature.^5^, ^19^, ^27^, ^29^, ^30^

An increase in *T*_2_* in the renal medulla after furosemide, indicating a decreased concentration of deoxyhemoglobin, has been reported by several other investigators^11^, ^13^, ^23^, ^24^, ^31^, ^32^ and has been ascribed to reduced oxygen consumption due to metabolic uncoupling of the sodium pump in the thick ascending limb of the loop of Henle. However, the possible influence of flow changes has not been addressed in human studies. Furosemide has additional effects on the kidney, including a fast onset and a volume independent increase of the renal sympathetic nerve activity.^33^ It is also well recognized that furosemide stimulates the renin‐angiotensin system, increasing plasma renin concentrations within as little as 10 minutes after injection of furosemide. Therefore, flow changes in the medulla as well as cortex, and metabolic changes due to changes of sodium load and distal sodium delivery are likely to occur, and hence may also influence *T*_2_*. The effect of furosemide stimulation on medullary perfusion has, to our knowledge, not previously been studied in humans, and results in animal studies have been conflicting. Brezis et al^34^, using oxygen electrodes and Doppler probes, noted a marked increase in medullary oxygenation despite a 26% decrease in medullary blood flow in rats. Kirchner^35^ found a decrease of medullary blood flow, but only in rats pre‐treated with indomethacin. Dobrowolski^36^ found a decrease in almost 50% of medullary blood flow in dogs within 10 minutes after furosemide injection, and this decrease was preventable by AT‐II blockade with saralasin or losartan. Likewise, Spitalewitz et al^37^ found a decrease in medullary blood flow (despite an increase in total renal blood flow) after furosemide that could be prevented by saralasin. We note that the doses of furosemide administered in the aforementioned animal studies were much higher ( ~4 mg kg^−1^ vs. ~0.3 mg kg^−1^) than the dose we have used.

Limitations of this study can be attributed to unknown physiological factors, technical issues and a lack of standardization in this area. When comparing MRI‐based measurements of the kidney, a standardized segmentation to define cortical and medullary tissue of the kidney is lacking, and a clear consensus is yet to emerge. This challenge is amplified given that the cortex is a relatively thin layer and that contrast between the outer and inner medulla (which have been shown in animal studies to react differently to certain stimuli) is poor. In this study, the main emphasis was on reproducibility and changes in measured values after furosemide injection. Thus, since the same ROIs were used before and after injection, and the same techniques for drawing ROIs and thresholding were used between Day 1 and Day 2, limiting the impact of ROI choice. A second technical challenge in this study is the use of multiple images in calculations requiring coregistration due to breathing. Other studies have evaluated the benefits of breath holding and respiratory gating to reduce movement,^38^, ^39^ though the reported improvements increase scanning time which would be a severe limitation in this study given the repeated interleaved measurements required. *T*_2_* has been validated to have a linear relationship to renal cortical and medullary tissue PO_2_ levels^40^, ^41^ and changes in *T*_2_* (or BOLD signal) have been validated as an indirect measure of changes in renal blood oxygenation.^7^, ^9^, ^42^ Still, shifts in factors such as arteriovenous shunting, plasma skimming and pH may alter the relationship between *T*_2_* and oxygenation.^12^ Lastly, physiological changes that vary from Day 1 to Day 2 or that are induced by furosemide could affect our measurements including hydration and sodium levels.

### 3.1 Perspectives

Measurements of renal cortical and medullary blood flow in combination with BOLD may be an important tool treating patient groups where complex interactions can be expected between renal perfusion and metabolism, such as CKD.^13^, ^43^, ^44^ In diabetic nephropathy, signs of hypoxia have been demonstrated in humans using MR‐BOLD in the renal cortex and medulla,^45^, ^46^ and hypoxia in the medulla and cortex increases in the initial stages of nephropathy before decreasing to levels lower than normal with increasing disease severity.^47^ This effect can perhaps be explained using simultaneous measurements of cortical and medullary perfusion. Further, these techniques can be combined with additional MRI techniques such as oxygen tension measures^48^ which may provide a more complete functional assessment and is clinically feasible. The pathogenesis of human essential hypertension is still unknown, but counteracting mechanisms that regulate medullary blood flow and oxygenation are believed to play a central role.^3^

In conclusion, using a contrast‐ and radiation‐free method, we have collectively measured changes in flow, perfusion and oxygenation in the renal cortex and medulla in humans. We could not only demonstrate the acknowledged increase in *T*_2_* in the medulla after injection of furosemide, but moreover confirm that it is not the result of changes in perfusion. The increase in *T*_2_* is, therefore, more likely ascribed to changes in oxygen tension due to decreased metabolism. The combination of perfusion, renal flow and *T*_2_* may be useful in the future research.

## 4 MATERIALS AND METHODS

### 4.1 Subjects

The study was approved by the local ethics committee, protocol H‐4‐2013‐132, and the participants gave informed written consent to participate according to the Helsinki declaration. Nine healthy adult subjects (age 20‐48 years, 6 males, BMI = 23.6 ± 2.9) with no history of high blood pressure and systolic blood pressure < 130 mmHg were included in the study.

### 4.2 Scanning protocol

The subjects were scanned on two occasions. All subjects were scanned between 6:00 AM and 12:00 PM, having fasted and thirsted from 11 PM the evening prior, to ensure similar degree of hydration with moderate activation of the urinary concentration mechanism. Scanning was performed on a 3‐T Philips Achieva scanner using the scanners body transmit and a four‐element SENSE cardiac receive coil. Subjects were scanned using MRI sequences to map ASL perfusion and BOLD *T*_2_* in the kidneys, and to measure blood flow in the renal artery. All three measurements were performed twice as shown in the scanning protocol in Figure 1. The scanning protocol was then repeated after the injection of 20 mg of furosemide. On a second day, subjects were scanned at the same time under the same conditions using an identical protocol, though the injection of furosemide and post injection scans were only repeated on a random subset of three subjects. Blood pressure (BP), blood oxygenation (SaO_2_) and heart rate (HR) were measured using a Veris Monitor system (MEDRAD, Pittsburgh, Pennsylvania, USA). The total scan time was approximately 35 minutes.

Prior to furosemide injection, subjects were scanned during a 5 minute handgrip exercise paradigm, known to instigate reductions in renal blood flow and perfusion using the same MRI measures as described in this procedure. Handgrip data from these subjects are also included in a larger published study where the complete hand grip intervention and data analysis have been described and the results analysed.^6^ Results for subjects in this study have not previously been reported as a separate subgroup.

### 4.3 Arterial spin labelling

ASL data were collected using a FAIR labelling scheme with a post‐label delay of 1100 ms and a balanced fast field echo (bFFE) readout scheme (TR 3.2 ms/TE 1.6 ms, flip angle 60^o^ SENSE factor 2 and linear acquisition).^5^ The matrix size was 144 × 144 covering a FOV of 288 mm × 288 mm with a 5 mm slice thickness. Acquisition parameters employed for acquiring ASL, *T*_1_ maps and *M*_0_ data are described in Gardener et al^38^ tag/control ASL pair was collected every 6 s with 30 ASL pairs acquired for each ASL measurement. A base equilibrium *M*_0_ scan and *T*_1_ map (*TI* values of 200‐1300 ms (100 ms steps) and 1500 ms) were acquired using respiratory triggering to allow ASL perfusion quantification.

After motion correction, the mean perfusion weighted difference of the control‐label pairs of images were quantified on a voxel‐by‐voxel basis, using the simplified perfusion model neglecting transit time effects and exchange time.^49^

$$f=\frac{\lambda }{2TI}\frac{\Delta M\left(\mathrm{TI}\right)}{M_{0}}exp\left(\frac{\mathrm{TI}}{T_{1}}\right)$$

where *M*_0_ is tissue equilibrium magnetization, *T*_1_ is the longitudinal relaxation in seconds, *f* is the perfusion rate in mL 100 g^−1^ min^−1^, and *λ* is the blood‐tissue partition coefficient, assumed to be 0.8 mL g^−1^ for kidneys.^20^

### 4.4 Transverse relaxation time, *T*_2_*

Single slice 2D multi‐echo fast‐field echo (mFFE) images were acquired using the same FOV and matrix as the ASL data with no SENSE factor, a TR of 59 ms and a flip angle of 10^o^. A total of 8 echoes were acquired per measurement, with an initial echo time (TE) of 1.4 ms and echo spacing of 8 ms steps. All 8 echoes were acquired in a single breath hold of approximately 25 seconds. *T*_2_* maps were calculated using a least squares fit minimizing to the equation:

$$S\left(\text{TE}\right)=S_{0}\;\times \;e^{-\text{TE}/{\text{T}}_{\text{2}}*}$$

### 4.5 Renal artery blood flow

Blood flow in the renal artery was calculated using phase contrast MRI (PC‐MRI). A single slice placed perpendicular to the renal artery of the right kidney was acquired using cardiac gating to image 20 time points of the cardiac cycle. Velocity encoding of 200 cm s^−1^, a TE/TR of 2.8/4.7 ms and turbo field echo acquisition were used with acquisition voxel dimensions of 2.5 × 2.5 × 8 mm reconstructed to 1.25 × 1.25 mm. All frames were acquired in a single breath hold of approximately 25 seconds. Renal artery blood flow was calculated from acquired images using Q‐flow software (Philips Medical Systems, Best), by selecting ROIs including the entire area of the renal artery cross section with each frame after correction for motion.

### 4.6 Data analysis

Reported values of cortical and medullary perfusion and *T*_2_* are mean values from ROIs drawn on both kidneys. ROIs were drawn to include as much as possible the entire volume of tissue excluding artefacts. Medullary ROIs were drawn to cover the innermost half of the medulla volume. All image manipulation, coregistration, calculations and statistical analysis were performed using scripts created in MATLAB 2013b (MathWorks, Natick, MA, USA).

### 4.7 Statistical analysis

Changes in parameters from the first two baseline measures and the two measures during intervention (handgrip exercise and after furosemide injection) were calculated and tested for significance using a two‐way ANOVA test with a threshold value of *P* < 0.05. Measurements are reported as mean ± standard deviation (SD). The coefficient of variation (CV) was calculated between measures on the first and second day. Handgrip data have been included in a separate study where repeatability and comparisons with physiological data are reported and discussed.^6^

## CONFLICTS OF INTEREST

The authors declare no competing financial interests.
